# Vitamin D Receptor Cdx-2 Polymorphism and Premenopausal Breast Cancer Risk in Southern Pakistani Patients

**DOI:** 10.1371/journal.pone.0122657

**Published:** 2015-03-23

**Authors:** Mehr un Nisa Iqbal, Taseer Ahmed Khan, Syed Amir Maqbool

**Affiliations:** 1 Department of Physiology, University of Karachi, Karachi, Pakistan; 2 Department of Clinical Oncology, Karachi Institute of Radiotherapy and Nuclear Medicine, Karachi, Pakistan; National Cancer Center, JAPAN

## Abstract

**Background:**

Vitamin D3 is a secoster oid that exerts its effect by binding to its nuclear receptor called vitamin D receptor (VDR), inducing apoptosis and thereby inhibiting cell proliferation in cancer cells. The VDR receptor, located in the nucleus, is known to regulate the functions of over 200 genes. Various allelic forms of hVDR have been discovered that increase susceptibility to various cancers. The VDR-Cdx2 polymorphism, located in the promoter region of exon 1e in the VDR gene, influences the functional activity of the receptor, since the hVDR lacks consensus TATA and CAAT boxes. The current investigation examines the association between VDR-Cdx2 polymorphism and breast cancer in premenopausal females from Southern Pakistan.

**Methods:**

We conducted a case control study on 264 subjects (103 cases and 161 controls) who were recruited from a tertiary hospital located in Karachi, Pakistan. Genomic DNA was extracted from peripheral blood using a commercial kit method, and the VDR-Cdx2 polymorphism was genotyped using tetraprimer amplification refractory mutation system polymerase chain reaction (T-ARMS-PCR) method. Pearson chi square test was used to assess the association between VDR-Cdx2 genotype and breast cancer while genotype distribution in controls was evaluated by Hardy-Weinberg equilibrium (HWE). Breast cancer risk was calculated using odds ratios and 95% confidence intervals.

**Results:**

The genotype distribution in the control group was in HWE (p > 0.05) for the VDR-Cdx2 polymorphism. A non-significant association was observed between VDR cdx2 polymorphism and breast cancer, however the GG genotype was at risk (OR = 1.832, 95% CI = 0.695–4.828) of developing breast cancer.

**Conclusion:**

The GG genotype of Cdx2-VDR gene polymorphism may increase the risk of developing breast cancer in young female patients in South Pakistan. Further investigations examining additional single nucleotide polymorphisms (SNPs) in VDR are required to assess their relationships with breast cancer.

## Introduction

In Pakistan, breast carcinoma is the most frequently diagnosed cancer and leading malignant disease. It is a major cause of death in Asian countries, accounting for one in five female patient deaths [1]. It is difficult to determine the epidemiology of breast carcinoma in the Pakistani population because the country lacks a functional national cancer registry system. However, Karachi, Pakistan, has the highest registered cases of breast carcinoma (38%) in female patients [2]^.^ Breast carcinoma is a multifactorial disease that is caused by various environmental [3] and genetic factors [4, 5]. There are numerous risk factors for breast cancer development, including excessive estrogen stimulus [6], elevated birth weight [7], overweight and obese [8], familial cancer history [9, 10] and gene mutations while mutation in BRCA 1 and BRCA 2 tumor suppressor genes are the most prevalent cause of breast cancer in Pakistani population [11–13]. However, limited data is available on tumor protein (TP53), phosphatase and tension homolog (PTEN), checkpoint kinase 2 (CHEK2), and estrogen Receptor 1(ESR) mutations involvement in the development of breast cancer. Pathogenesis of breast cancer has not been well characterized [14, 15], but data have been published demonstrating the defensive role of 1,25 (OH)2 vitamin D3 in certain cancers [16–18].

Vitamin D3 has anti-cancer activity, and the deficiency may affect breast cancer vulnerability since vitamin D plays an important role in regulation of cell growth, cell proliferation, cell differentiation and cell death [19]. A previous study on Pakistani population has reported 95.6% of breast cancer patients along with 77% of healthy females as being vitamin D deficient [20] which is in contrary to the findings in developed countries.

Vitamin D is known to mediate its action in an intracrine or autocrine manner via binding to its receptor VDR [21, 22] which is expressed in most body tissues as well as on cancerous cells. The vitamin D receptor gene is a broadly studied candidate gene that resides in chromosome 12q region 14, consisting of a 5’ non translated promoter region and 5 exons. The DNA binding domains are encoded by exons 1A, 1B and 1C along with exons 2 and 3, whereas the ligand binding domains are encoded by exons 4 and 5 [23]. It has been previously demonstrated that VDR expression is reduced in cancer cells [24], which may be influenced by polymorphisms within the gene and increase breast cancer incidence [25]. Over 200 polymorphisms have been discovered in the VDR gene in different individuals, and Cdx2, Fok1, Apa1, Taq1 and Bsm1 are the most commonly studied polymorphisms. Several studies have been carried out to evaluate the relationship between VDR gene polymorphisms and breast cancer risk [26–29]. A meta-analysis on Caucasian women has demonstrated that VDR (Fok1, Bsm1, Apa1 and Taq1) polymorphisms may not be associated with increase breast cancer susceptibility [30] which is in contrary to the findings of Zhang and Song [31] in which a strong association between FokI VDR polymorphism and breast cancer has been reported. Others have also highlighted Fok1 SNP as the most notorious polymorphism of VDR gene influencing breast cancer susceptibility [25, 32, 33]. Among all VDR polymorphisms limited data is available on VDR-Cdx2 polymorphism and breast cancer susceptibility.

Cdx2 which is an intestine-specific transcription factor is known to regulate proliferation and differentiation of cells. Cdx2 is generally a protective polymorphism, particularly in osteoporosis [34, 35]. However, the published data examining the relationship between VDR Cdx2 variants and breast cancer susceptibility have been contradictory [25, 27, 36, 37].

The polymorphism of Cdx2 in VDR gene is located in the exon 1 promoter region of VDR gene and is a guanine/adenine (G/A) sequence variation [38]. The frequency of the A allele is 74% in Africans, 43% in Asians and 19% in Caucasians [39].The A allele allows the Cdx2 transcription factor to bind more effectively, thereby increasing VDR transcriptional activity thus decreasing the breast cancer incidence [40], whereas the G allele has the opposite effect [34,36]. The CDx2 AG genotype has been reported to decrease breast cancer risk, whereas the AA genotype increases this risk [27]. Another study observed that the A allele may increase bone mineral density by increasing intestinal VDR expression, allowing for increased intestinal calcium absorption [34, 41]. Conversely, a study observed no correlation between the VDR Cdx2 polymorphism and breast cancer incidence [37].

Undeniably, adequate research has not been performed to determine the potential link between Cdx2 and breast cancer development in Pakistan. The linkage disequilibrium (LD) pattern between the Cdx2 and other VDR polymorphisms has not been studied with respect to breast cancer risk [37]. In conclusion, there are no data concerning the VDR Cdx2 polymorphism in the Pakistan population.

## Materials and Method

### Ethics Statement

The study was approved by the Board of Advanced Studies and Research (Approval#10(27)28032012), University of Karachi and the Ethical Review Board of Departmental Research Committee (DRC), Department of Physiology, University of Karachi prior to its commencement. Written informed consent was obtained from all subjects prior to sample collection.

### Study population

The present investigation is a retrospective, cohort case (n = 103) control (n = 161) study performed in pre-menopausal subjects with documented breast cancer diagnosis based on histopathology results. Subjects were recruited from September 2012 to July 2014 at a tertiary hospital located south of Karachi, while control subjects were healthy volunteer females selected randomly having no prior history of cancer. Inclusion criteria: Subjects between the ages of 18 and 45 years, parous, nulliparous and premenopausal, with or without a positive family history of breast cancer were included. All other subjects were excluded.

### DNA extraction

Genomic DNA was extracted from peripheral blood using a commercial kit method (Gene JET Genomic DNA Purification Kit, Thermo Fisher Scientific Baltics UAB, Vilnius, Lithuania). Briefly, 200 μl whole blood, 400 μl R.B.C lysis solution and 20 μl proteinase K were mixed in a 1.5 ml eppendorf tube and incubated at 56°C for 10 min, followed by the addition of 500 μl 96% ethanol. Subsequently, whole lysate was pipetted into a DNA purification column and centrifuged at 13000 rpm for 1 min at 4°C. The column was washed with 500 μl of wash buffers I and II for 1 min each at 13000 rpm. Genomic DNA was eluted using 100 μl elution buffer and stored at -86°C for further investigation.

### PCR analysis

The Cdx2 VDR gene polymorphism (rs 11568820) was genotyped using the polymerase chain reaction method as described earlier [42]. The G-Forward (5ʹ-AGGATAGAGAAAATAATAGAAAACATT-3ʹ) and Reverse (5ʹ-AACCCATAATAAGAAATAAGTTTTTAC-3ʹ) primers, specifically amplify the G allele, with a product size of 110 bp. The A-Forward (5ʹ-TCCTGAGTAAACTAGGTCACAA-3ʹ) and Reverse (5ʹ-ACGTTAAGTTCAGAAAGATTAATTC-3ʹ) primer pairs specifically amplify the A allele, with a product size of 235 bp. A 297 bp internal control was amplified using the G-Forward and A-Reverse primers. The reaction was performed in a total volume of 50 μl using KapaTaq DNA polymerase master mix (KapaBiosystem, USA). Briefly, 25 μl of 2x master mix, 0.3 μl of each primer pair (25 pmol), 3 μl of genomic DNA and 20.2 μl of water were amplified in an automated thermal cycler (Veriti, Applied Biosystem, USA) under the following conditions: Initial denaturation at 95°C (5 min), 40 cycles of 95°C (30 sec), 56°C (45 sec), and 72°C (45 sec), followed by a final extension at 72°C for 5 min. The amplicons were electrophoresed with a 100 bp ladder (Fermentas, USA) on an 8% polyacrylamide gel at 30 mA for 1 hr, followed by staining with 0.5 μg/ml ethidium bromide. Gel images were obtained using the ChemiDoc-It2 (UVP, UK) Vision works LS software (version 7.1).

### Statistical Analysis

Statistical analysis was performed using SPSS (version 17), with statistical significance set at p<0.05. Hardy-Weinberg equilibrium (HWE) was evaluated by Chi-square test in control. We used unconditional logistic regression to calculate the odds ratios with 95% confidence interval. Pearson chi-square (χ^2^) and odd ratios with 95% confidence intervals were calculated to assess the strength of association between VDR Cdx2 polymorphism and breast cancer risk. Odds ratios were also calculated for allele frequency comparison (G vs A), homozygote comparison (GG vs AA), dominant model (AA+GA vs GG), and recessive model (GG+GA vs AA).

## Results and Discussion

Breast cancer prognosis in Pakistan remains uncertain because the country lacks a cancer registry system. This was a major impediment to the present study because reliable disease prevalence data could not be obtained. Breast cancer remains the most threatening and devastating cancer among women throughout the world, particularly in Pakistan. However, its pathogenesis, similar to other cancers, remains unclear. Several risk factors have been highlighted, including genetic predisposition [14, 15, 43]. Lifestyle remains a major contributor to breast cancer onset. Vitamin D, synthesized in the skin after sun exposure, and its metabolites (D3) have been reported to have an inverse relationship with breast cancer risk [44, 45]. However, vitamin D3 stimulates the transcription of multiple genes involved in cellular differentiation via its receptor, VDR, [44] located in the cell nucleus. VDR expression is influenced by single nucleotide polymorphisms (SNPs) [46, 47]. Over 200 SNPs have been identified in VDR; however, the polymorphisms most commonly associated with tumorigenesis are Fok 1 (ff) [48], Bsm 1 (bb) [49], Apa 1 (AA) [50], and Cdx2 (AA) [36], with Cdx2 being the least studied. To date, no data have been reported concerning the association between VDR polymorphisms and breast cancer in the Pakistani population. Therefore, we examined the risk and association between the VDR-Cdx2 polymorphism and breast cancer in Southern Pakistani females. It was previously identified that the structure of VDR gene is divided in three basic regions: 5’ untranslated (UTR) promoter region (Cdx2), coding regions (Fok 1, Bsm 1, Apa 1, Taq 1, Tru 91) and 3’ UTR regulatory region (poly A tail) because Cdx2 lie in 5’ UTR promoter region that’s why we considered only this SNP for evaluating the association with breast cancer risk.

This case control study consisted of 103 patients and 161 healthy controls with a mean age of 35±6 years. The breast cancer-related clinical characteristics of the study population are summarized in Table 1. The distribution of ER+/-, PR+/- and Her2neu+/- among patients was 1:0.23, 1:0.25 and 1:0.06, respectively. These factors have historically been employed to aid in the clinical management of breast cancer [51]. The majority of cases were invasive ductal carcinoma (91%) with disease stage grade II (56%) and III (33%), however a few cases of invasive papillary carcinoma (3%), ductal carcinoma in situ (DCIS, 2%) and others was also observed. Unfortunately, in Pakistani society, breast cancer cases are identified at later stages because of societal inhibitions, despite the disease being curable when identified at an early stage.

**Table 1:** Breast cancer clinical characteristics.

Clinical Characteristics	Cases (n = 97)
Type of Breast cancer	
Invasine ductal carcinoma	88
Invasive Papillary Carcinoma	3
Invasive ductal carcinoma in situ (DCIS)	2
Others	4
Grades of cancer	
GI	6
GII	54
GIII	32
GIV	5
Estrogen receptor status	
ER+ve (0 to +9)	34
ER-ve (-1 to-2)	8
Progesterone receptor status	
PR+ve (+0 to +8)	31
PR-ve (-1 to-2)	8
Her 2 neu (erb 2 oncoprotein)	
+ve (0 to +3)	31
-ve (-1 to-2)	2

The breast cancer risk factors and anthropometric characteristics of patients and controls are depicted in Table 2. This study showed that the breast cancer risk significantly increases with increasing body weight (BMI > 25) and increasing waist to hip ratio (WHR > 0.85) whereas early menarche (< 12 yrs), lengthy menstrual phase, first birth at late ages (> 25 yrs), positive family history of breast cancer and all subjects who took oral contraceptives (OC) and receiving hormone replacement therapy (HRT) may also be at higher risk of breast cancer but these findings were not statistically significant. On contrary, other factors (nulliparity, no lactational history and positive family history of other cancers) were not associated with the breast cancer risk.

**Table 2:** Risk factors associated with the Breast cancer.

Factors	Cases n	Controls n	χ2	P-value	OR (95%CI)*	P-value
Menarcheal age						
>14	9	11	2.347	0.309	1	
12_14	88	146			0.737 (0.294–1.848)	0.515
<12	6	4			1.833 (0.392–8.566)	0.441
Cycle length						
Normal (3–7)	95	141	3.606	0.165	1	
Short (1–2)	3	14			0.318 (0.089–1.137)	0.078
Long (> 7)	5	6			1.273 (0.3674–4.169)	0.732
Parity						
Parous	85	25	122.262	0.000	1	
Nulliparous	18	136			0.034 (0.017–0.067)	0.000
Age at first birth						
<20	22	3	1.118	0.773	1	
20–24	34	7			0.711 (0.114–4.442)	0.715
25–29	16	5			1.079 (0.19–6.117)	0.931
>29	9	2			1.63 (0.232–11.455)	0.624
Lactational history						
Yes	83	23	112.407	0.000	1	
No	20	138			0.042 (0.022–0.081)	0.000
BMI						
Normal (18–25)	42	82	45.717	0.000	1	
Underweight (< 18)	7	52			0.263 (0.110–0.629)	0.003
Overweight (25–30)	39	20			3.807 (1.978–7.328)	0.000
Obesity (> 30)	15	7			4.184 (1.584–11.04)	0.004
W/H ratio						
Acceptable (<0.85)	11	85	45.789	0.000	1	
Uacceptable (>0.85)	92	76			8.139 (4.211–15.732)	0.000
Family history						
No	81	131	0.295	0.587	1	
Yes	22	30			1.186 (1.384–5.876)	0.587
F.H of other cancer						
No	88	131	0.273	0.601	1	
Yes	15	30			0.837 (0.429–1.632)	0.602
O.C						
No	93	148	0.211	0.646	1	
Yes	10	13			1.224 (0.516–2.905)	0.646
H.R.T						
No	98	158	1.912	0.167	1	
Yes	5	3			2.687 (0.628–11.494)	0.183

*Odds ratios and 95% CI was calculated by logistic regression analysis

Other factors that modify the breast cancer such as grade, ER/PR status and Her2 neu status of breast cancer which were determined and difference were observed among cases. Low grade and ER+ve exhibited slightly higher risk of carrying GG genotype but these findings were not significant statistically (Table 3).

**Table 3:** Association of AA and GG genotype in relation to its covariates.

Variables	n	GG+GA	AA	*OR (95%CI)	P-Value	AA+GA	GG	*OR (95%CI)	P-Value
Grades of cancer									
GI	6	5	1	1		3	3	1	
GII	54	51	3	0.294 (0.025–3.383)	0.326	30	24	1.38 (0.268–7.153)	0.697
GIII	32	29	3	0.571 (0.044–6.019)	0.598	13	19	0.45 (0.09–2.244)	0.33
GIV	5	5	0	0 (NC)	0.999	3	2	0.5 (0.048–5.154)	0.56
Estrogen receptor status									
ER-ve (-1 to-2)	8	8	0			4	4	1	
ER+ve (0 to +9)	34	34	0	NC**		16	18	1.125 (0.241–5.252)	0.881
Progesterone receptor status									
PR-ve (-1 to-2)	8	8	0			3	5	1	
PR+ve (+0 to +8)	31	31	0	NC**		14	17	0.729 (0.148–3.596)	0.697
Her 2 neu									
-ve (-1 to-2)	2	2	0			0	2	1	
+ve (0 to +3)	31	31	0	NC**		12	19	0 (NC**)	0.999

*Unconditional logistic regression analysis

**Not calculated

The genotype distribution of GG (53), GA (37), and AA (7) among breast cancer patients was obtained in 97 samples out of 103; however, in controls, it was 62, 84 and 15 respectively. The genotype distribution of control was in Hardy-Weinberg equilibrium with p-value 0.07 (Table 4). Our results also demonstrated no-association (Pearson’s χ^2^ = 0.032, p-value = 0.984) between the Cdx2 polymorphism and breast cancer, while an odds ratio via binary logistic regression analysis of 1.832 (95%CI = 0.695–4.828) was observed for the GG genotype (Table 5). The observed allele frequency difference was non-significant (G vs A allele, P = 0.109; OR = 1.578; 95%CI = 0.903–2.758). An odds ratio for GG vs AA model is 1.832 (95%CI = 0.695–4.828), recessive model (GG+GA vs AA) OR = 1.321 (95%CI = 0.519–3.364) and dominant model (AA+GA vs GG) OR = 0.754 (95%CI = 0.453–1.257) were non-significant.

**Table 4:** Genotype distribution in the Hardy-Weinberg equilibrium.

Genotype	Observed value (n)	Predictive value (n)	χ2	p-value
GG	62	67.2	3.18	0.07
GA	84	73.6		
AA	15	20.2		

**Table 5:** Association between VDR Cdx2 polymorphism and BC risk.

Genotype	Cases	Controls	χ2	p-value	*OR (95% CI)	p-value
	(n)	(n)				
AA	7	15	0.032	0.984	1	
GA	37	84			0.944 (0.355–2.507)	0.908
GG	53	62			1.832 (0.695–4.828)	0.221
Total	97	161				
A	25.5	57			1	
G	71.5	104			1.578 (0.903–2.758)	0.109
GG vs AA	53/7	62/15			1.832 (0.695–4.828)	0.221
GG+GA vs AA	90/7	146/15			1.321 (0.519–3.364)	0.559
AA+GA vs GG	44/53	99/62			0.754 (0.453–1.257)	0.279

*Unconditional logistic regression analysis

**Fig 1:**
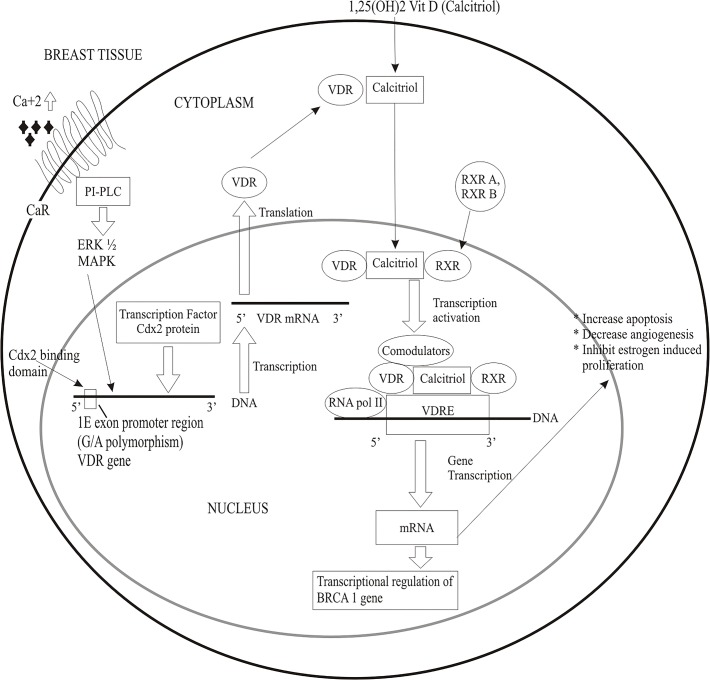
Signaling pathway indicating the relationship between Cdx2 VDR and breast cancer. See text for further details. CaR = Calcium receptors, PI-PLC = Phosphotidyl inositol—phospholipase C, ERK ½ = Extracellular signal regulated kinases 1 and 2, MAPK = Mitogen activated protein kinases, RXR A and B = Retinoid X receptor A and B, VDRE = vitamin D receptor response element.

The results from our study revealed a non-significant association between VDR-Cdx2 gene polymorphism and breast cancer among premenopausal Pakistani females, while the published findings on the relationship between VDR Cdx2 polymorphisms and breast cancer incidence are conflicting [27, 36, 38] since a significant association has been documented in an African population [27] only. However a study on colorectal cancer risk [52] and prostate cancer [53,54] with VDR Cdx2 has reported a significant and non-significant association respectively. Our results showed an odds ratio (OR) for VDR-Cdx2 G allele as 1.578 (95%CI = 0.903–2.758, p = 0.109), which is in agreement with previous study [34] who had identified that Cdx2-G allele is responsible to lower the transcriptional activity of VDR gene. The decrease in transcriptional activity of VDR alters the various protective mechanism regulated by vitamin D3 (Fig. 1). Studies have documented that Cdx2 may promote tumor development [55, 56, 57], while other studies have identified it as a tumor suppressor [58, 59, 60]. Since VDR-Cdx2 domain regulates the expression of VDR gene in mammary tissue as the human VDR promoter domain lacks consensus TATA and CAAT boxes [61]. Therefore the homeo domain may play a vital role in regulating tumor suppressor gene (BRCA1 gene) [62] and prevents estrogen induced ductal proliferation and differentiation [63, 64] leading to prevention in breast cancer development. Additionally vitamin D through VDR induces apoptosis in breast tissue via down regulation of the anti-apoptotic protein (Bcl-2) [18] and inhibition of angiogenesis in mammary tissues [65, 66].

### Strengths and Limitations

The study has the strength that it attempts to answer the probable contribution of vitamin D receptor genes in breast cancer among premenopausal Pakistani females. Since a significant haplotype differences between cases and controls may play a role in influencing vitamin D supplementation to improve health status of breast cancer subjects.

Present investigation has the limitation of relatively small sample size along with the single SNP of VDR gene. These limitations could be improved by enrolling a greater number of individuals, studying additional VDR-SNPs, evaluating serum vitamin D3 levels, and assessing various clinicopathological parameters and other risk factors. However, this study is the first performed in pre-menopausal breast cancer cases in South Asia, specifically in Pakistan.

## Conclusions

The GG genotype of VDR-Cdx2 polymorphism may increase the risk of breast cancer in females from Karachi, Pakistan. The present study should prompt further large-scale studies of premenopausal breast cancer predisposition genes in larger sample sizes and the collection of additional demographic data related to ethnicity, diet, lifestyle, reproductive hormone levels, oral contraceptive usage and other environmental factors.
